# Protein *O*-Fucosyltransferase 2 Is Not Essential for *Plasmodium berghei* Development

**DOI:** 10.3389/fcimb.2019.00238

**Published:** 2019-07-03

**Authors:** Silvia Sanz, Eleonora Aquilini, Rebecca E. Tweedell, Garima Verma, Timothy Hamerly, Bernadette Hritzo, Abhai Tripathi, Marta Machado, Thomas S. Churcher, João A. Rodrigues, Luis Izquierdo, Rhoel R. Dinglasan

**Affiliations:** ^1^ISGlobal, Barcelona Centre for International Health Research (CRESIB), Hospital Clínic-Universitat de Barcelona, Barcelona, Spain; ^2^Department of Molecular Microbiology and Immunology, Johns Hopkins Bloomberg School of Public Health, Baltimore, MD, United States; ^3^Department of Infectious Diseases and Immunology, The University of Florida Emerging Pathogens Institute, Gainesville, FL, United States; ^4^Instituto de Medicina Molecular, Unidade de Malária, Universidade de Lisboa, Lisbon, Portugal; ^5^Department of Infectious Disease Epidemiology, MRC Centre for Outbreak Analysis and Modelling, Imperial College London, London, United Kingdom

**Keywords:** *Plasmodium falciparum*, *Plasmodium berghei*, *O*-fucosylation, protein *O*-fucosyltransferase 2, oocyst, sporozoite

## Abstract

Thrombospondin type I repeat (TSR) domains are commonly *O*-fucosylated by protein *O*-fucosyltransferase 2 (PoFUT2), and this modification is required for optimal folding and secretion of TSR-containing proteins. The human malaria parasite *Plasmodium falciparum* expresses proteins containing TSR domains, such as the thrombospondin-related anonymous protein (TRAP) and circumsporozoite surface protein (CSP), which are *O*-fucosylated. TRAP and CSP are present on the surface of sporozoites and play essential roles in mosquito and human host invasion processes during the transmission stages. Here, we have generated PoFUT2 null-mutant *P. falciparum* and *Plasmodium berghei* (rodent) malaria parasites and, by phenotyping them throughout their complete life cycle, we show that PoFUT2 disruption does not affect the growth through the mosquito stages for both species. However, contrary to what has been described previously by others, *P. berghei* PoFUT2 null mutant sporozoites showed no deleterious motility phenotypes and successfully established blood stage infection in mice. This unexpected result indicates that the importance of *O*-fucosylation of TSR domains may differ between human and RODENT malaria parasites; complicating our understanding of glycosylation modifications in malaria biology.

## Introduction

Malaria is one of the most important human parasitic diseases, causing ~219 million new cases and more than 400,000 deaths every year (WHO, 2018). It is caused by a protozoan apicomplexan parasite of the genus *Plasmodium*, with *Plasmodium falciparum* regarded as the deadliest species. *Plasmodium* parasites are transmitted by female *Anopheles* mosquitoes. After the bite of an infected mosquito, motile sporozoites are injected into the human dermis; from there, they travel through blood vessels to the liver and infect hepatocytes. A week later, the infected hepatocyte ruptures and releases merozoites that reach the blood circulation and invade erythrocytes initiating cyclical asexual reproduction. A small percentage of blood stage parasites become sexually committed cells known as gametocytes, which are taken up during a mosquito blood meal. Once in the mosquito midgut, gametocytes develop into gametes, fertilization takes place, and zygotes are formed. These zygotes develop into motile ookinetes that traverse the mosquito midgut wall to form oocysts. Each oocyst can develop into thousands of sporozoites that invade the mosquito salivary glands and are ready to infect another human host (Menard et al., 2013).

Thrombospondin type 1 repeat (TSR) domains are small (50–60 amino acid residues) cysteine-knot motifs with 3 conserved disulfide bonds that play important roles in cell adhesion and motility (Adams and Tucker, 2000; Tan et al., 2002). *Plasmodium* parasites express several TSR domain-containing proteins throughout the different stages of their life cycle that are critical for host cell recognition, motility, and invasion (Morahan et al., 2009). These proteins include circumsporozoite protein (CSP) and thrombospondin-related anonymous protein (TRAP) in the sporozoite stage and circumsporozoite and TRAP-related protein (CTRP) in the ookinete stage (Wengelnik et al., 1999; Coppi et al., 2011; Mathias et al., 2013). Antibodies against these proteins inhibit host cell invasion and block the progression of the parasite's life cycle (Chattopadhyay et al., 2003; Li et al., 2004). Hence, TSR domain-containing proteins are potentially important vaccine targets (Moorthy et al., 2004). Indeed, the licensed malaria vaccine RTS,S (Mosquirix) is based on CSP (RTS,S Clinical Trials Partnership, 2015).

TSR domains are *O*-fucosylated by protein *O*-fucosyltransferase 2 (PoFUT2) (Luo et al., 2006; Leonhard-Melief and Haltiwanger, 2010). That fucose can be further elongated with a glucose residue, generating an *O*-linked disaccharide (Kozma et al., 2006). This modification is important for the secretion of TSR domain-containing proteins (Ricketts et al., 2007; Wang et al., 2007; Vasudevan and Haltiwanger, 2014). A recent report demonstrated that CSP and TRAP TSR domains are also *O*-fucosylated in the *Plasmodium* sporozoite stage (Swearingen et al., 2016). A homolog of PoFUT2 is conserved in all the *Plasmodium* species sequenced (Cova et al., 2015), and the parasite synthesizes the GDP-fucose (GDP-Fuc) precursor required for *O*-fucosylation (Sanz et al., 2013, 2016; López-Gutiérrez et al., 2017). In a recent study, Lopaticki et al. (2017) characterized the *P. falciparum* protein *O*-fucosyltransferase (PoFUT2) and showed that it is involved in the *O*-fucosylation of parasite TSR domains. The authors reported that PoFUT2 genetic disruption in *P. falciparum* resulted in a reduction in the ability of ookinetes to traverse the mosquito midgut to form oocysts. They also provided evidence showing that mosquitoes infected with ΔPoFUT2 parasites harbored significantly fewer sporozoites in the mosquito salivary glands compared to mosquitoes infected with the wild type *P. falciparum* parental line, NF54. Finally, they assessed the infectivity of salivary gland sporozoites by analyzing their motility, cell traversal activity, and hepatocyte invasion and by carrying out co-infection experiments using wild type and mutant parasites in a humanized chimeric liver mouse model; these assays revealed an apparent lower fitness of ΔPoFUT2 parasites in completing development in the mosquito and infecting mammalian hepatocytes compared to wild type NF54. Here, we report a robust study that differs from the central results of the previous report and reveals that under laboratory conditions, that PoFUT2 is not essential for murine parasite development and transmission.

## Materials and Methods

### Ethics Statement

The human blood used for mosquito blood meals and *P. falciparum* culture was collected from a pool of pre-screened donors under an IRB-approved protocol at Johns Hopkins University (Protocol NA00019050) or obtained commercially from anonymous donors through Interstate Blood Bank or Banc de Sang i Teixits (Catalonia, Spain), after approval from the Comitè Ètic Investigació Clínica Hospital Clínic de Barcelona, making informed consent not applicable. Animal experiments were approved by the Portuguese official veterinary department for welfare licensing and the Instituto de Medicina Molecular Animal Ethics Committee. All animal experiments were performed in strict compliance to the guidelines of the institution's animal ethics committee and the Federation of European Laboratory Animal Science Associations (FELASA).

### *P. falciparum* Asexual Parasite Culture and Transfection

*P. falciparum* NF54 parasites (*Pf*WT) (a kind gift of Teun Bousema, Radboud University Nijmegen Medical Center) were cultured with human B^+^ erythrocytes (2–4% hematocrit) in complete culture medium (RPMI media [Sigma] supplemented with 10% AB^+^ human serum or 0.5% Albumax II), incubated at 37°C in an atmosphere of 92% N_2_, 3% O_2_, and 5% CO_2_ using standard methods (Trager and Jensen, 1976). *Pf*WT parasites were transfected by schizont nucleofection as described previously (Moon et al., 2013). After the appearance of resistant parasites, on/off drug cycling with WR22910 using 2-week cycles was used, followed by negative selection with 5-fluorocytosine (5FCyt) to select double recombinant parasites. Integrant clonal parasites were obtained by limiting dilution.

### *P. falciparum* Transfection Construct

**Figure 1:**
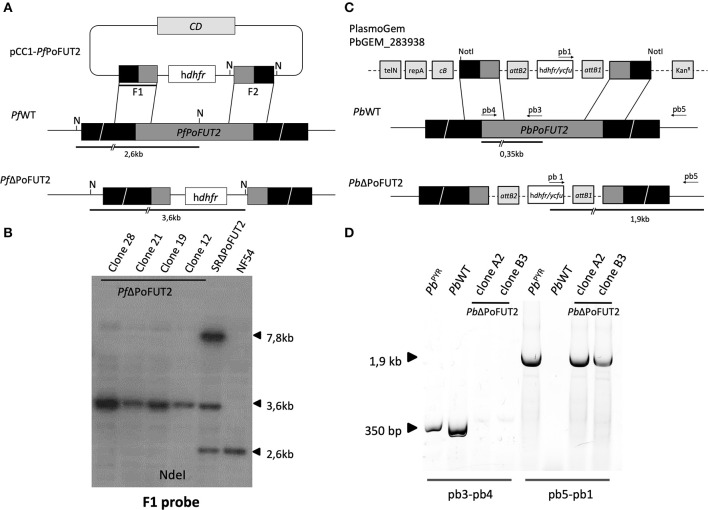
PoFUT2 transfection constructs and integration events in human and rodent malaria parasites. **(A)** Scheme of the transfection plasmid (pCC1-*Pf*PoFUT2) used to target and disrupt *Pf*PoFUT2 in *P.falciparum* NF54 parasites (*Pf*WT) and the expected double crossover recombination event (*Pf*ΔPoFUT2). Black boxes represent upstream and downstream DNA sequence flanking the *Pf*PoFUT2 gene locus. Position of NdeI (N) sites, position of F1 probe (thick black line) and predicted length of the restriction fragments (thick black lines) are shown. **(B)** Southern blot of NdeI digested genomic DNA from *Pf*WT, SRΔPoFUT2, and *Pf*ΔPoFUT2 parasite clones hybridized with the F1 probe. SR refers to single recombinant parasites before negative selection with 5-FCyt. A 7.8Kb band (size of the pCC1-*Pf*PoFUT2 plasmid) can be also observed in SRΔPoFUT2 lane. **(C)** Scheme of the PlasmoGem transfection plasmid (PbGEM-283938) used to disrupt *Pb*PoFUT2 in *Pb*WT parasites and the expected double crossover recombination events (*Pb*ΔPoFUT2). Black boxes represent upstream and downstream DNA sequence flanking the *Pb*PoFUT2 gene locus. Position of NotI, predicted length of PCR fragments (black lines), and position of primers pb1, pb3, pb4, and pb5 are shown. TelN refers to telomerase, RepA to helicase, *cB* to arabinose-inducible origin, and Kan^R^ to the kanamycin resistance cassette. *att*B1 and *att*B2 are the recombination sequences for the Gateway technology. **(D)** Genotype analysis of *P. berghei* transfectants. PCR using genomic DNA from *Pb*WT, pyrimethamine-resistant population (*Pb*^PYR^) and *Pb*ΔPoFUT2 clones as template were done using primers pb3 and pb4, and pb5 and pb1for wild type locus and integration (*Pb*PoFUT2 disruption), respectively.

pCC1-*Pf*PoFUT2 transfection construct (Maier et al., 2006) consisted of two fragments of ≈ 850 and ≈ 960 bp, respectively, from different regions of the *Pf*PoFUT2 locus. F1 (nucleotides from −361 to +479 of the *Pf*PoFUT2 locus) and F2 (nucleotides from +1363 to 630 after the *Pf*PoFUT2 locus) fragments were amplified with primers pf3 (CAATGGCCCCTTTCCGCGGTCTTATGTCTTATTCTCATTTTGCTT) and pf4 (AGATCTTCGGACTAGTCTTTTTGTAGCTGCAAGGGGG) for fragment F1, and pf5 (ATCGATAACTCCATGGTGAGCAATGGATTTGTACAAGGT) and pf6 (CAGGCGCCAGCCTAGGTCAAGTGCAAGGGTTCTTTT) for fragment F2. The construct generated would integrate into the PoFUT2 locus, disrupting it by double crossover homologous recombination (Figure 1A).

### *P. falciparum* Southern Blotting and PCR Analysis

Two μg of genomic DNA (gDNA) from *Pf*WT, SRΔPoFUT2 (single recombinant parasites before 5FCyt selection), and *Pf*ΔPoFUT2 (*P. falciparum* PoFUT2 null mutant; four different clones) were digested with NdeI and probed with ^32^P (Perkin Elmer)-labeled F1 (Figure 1B).

### Mice

BALB/c and C57BL/6 mice (6–8 weeks of age) were purchased from Charles River and housed in the rodent facility of Instituto de Medicina Molecular (Lisbon, Portugal).

### *Plasmodium berghei* Transfection, Cloning, and PCR Genotyping

Transfection experiments were performed using *Plasmodium berghei* ANKA strain 2.34 parasites (Janse et al., 2006). The PoFUT2 knockout vector PbGEM-283938 was obtained from the PlasmoGEM resource (Pfander et al., 2011) (http://plasmogem.sanger.ac.uk). The final knockout construct was digested with NotI to release the fragment for transfection (Figure 1C). The pyrimethamine-resistant parasite population (Pb^PYR^) containing the correct genomic integration substituting PoFUT2 gene (*Pb*ΔPoFUT2) was cloned by injecting one parasite per mouse (BALB/c male mice, 6–8 weeks of age) to obtain *Pb*ΔPoFUT2. *hu-dhfr* cassette integration conveying resistance to pyrimethamine was tested using primers pb1 (CATACTAGCCATTTTATGTG), pb3 (AGCACCACGGGGGAAGGACT), pb4 (ATGCAAAAACGTCTTCCCTT), and pb5 (TCGAGCAACGATAAAATGCCT) (Figure 1D).

### *P. falciparum* Gametocyte Cultures and Mosquito Infection

*Pf*WT and *Pf*ΔPoFUT2 were diluted to 0.5% mixed stage asexual parasites and 4% hematocrit with complete culture media and cultured at 37°C using the candle jar method (Trager and Jensen, 1976). The media was exchanged daily from day 1 to day 17 to allow for gametocyte maturation from stage I through stage V. Standard membrane feeding assays (SMFA) were performed on day 15–18 post-culture initiation. Approximately 60 female *An. stephensi* (SDA-500) or *An. gambiae* (KEELE) mosquitoes were distributed into pint-sized cups and starved of sugar and water for ≈ 12 h prior to feeding. *Pf*WT and *Pf*ΔPoFUT2 gametocyte cultures were pelleted and diluted to 0.03 or 0.3% gametocytemia with human blood at 50% hematocrit. Blood was washed with RPMI media and brought to 50% hematocrit with heat-inactivated AB serum. Gametocytemic blood was kept at 37°C until feeding. Approximately 250–300 μL of gametocytemic blood was dispensed into a water-jacketed membrane feeder at 37°C, and mosquitoes were allowed to feed for a minimum of 45 min. After blood feeding, non-blood fed mosquitoes were removed. Blood fed mosquitoes were kept at 26°C and 70% humidity with a 12-h light:dark cycle. Mosquitoes were provided a 10% sucrose solution for energy.

### *P. berghei* Mosquito Infections

*An. stephensi* mosquitoes were bred at the insectary of the Instituto de Medicina Molecular. For mosquito infection, female BALB/c mice were intraperitoneally injected with *P. berghei* wild type (*Pb*WT) and *Pb*ΔPoFUT2 mutant lines. Three to 5 days post-infection, the number of exflagellation events was determined using a Zeiss Axioskop 2 light microscope and a counting grid. If > 1 exflagellation event per field of view was observed, mice were anesthetized with a mixture of 10% ketamine and 2% xylazin in phosphate buffered saline (PBS) (100 μL per 20 g mouse body weight i.p.) and fed to *An. stephensi* mosquitoes. Unfed mosquitoes were removed, and fed mosquitoes were maintained at 19–22°C in 50–80% relative humidity. Mosquitoes were used 10–23 days post infection for further experiments.

### Oocyst Counting and Imaging

For *P. falciparum* oocyst counting, mosquito midguts were dissected 8 to 10 days post-feeding; midguts were stained with 0.2% mercurochrome in water for 9 min. Midguts were placed on a slide with a drop of PBS, overlaid with a coverslip, and examined for oocysts using brightfield microscopy at 200× total magnification. Each midgut was imaged with ProGres CapturePro software to measure oocyst diameter, ensuring oocyst in all planes were visible. Six biological replicates with two technical replicates each were analyzed. *P. berghei* infected midguts were collected and stained with 0.5% mercurochrome 10 days post-feeding. Oocysts were counted to determine the intensity of infection (number of oocysts per midgut). Twelve experiments were carried out, and generalized mixed effect models (GLMM) were used for or statistical analysis.

### Sporozoite Purification and Counting

On day 14 post-feeding, 30 mosquitoes infected with each *P. falciparum* line (*Pf*WT and *Pf*ΔPoFUT2) were dissected to obtain salivary glands. Each pair of salivary glands was kept in 100 μL PBS in a 1.5 mL tube. The tubes were spun at 1,200 × g for 3 min at room temperature (RT). The salivary gland pellet was gently crushed and vortexed for 3 s to resuspend the salivary gland contents. The tubes were spun again and sporozoites were counted blindly on a Zeiss Axioskop 2 microscope using a hemocytometer and averaging counts from 2 fields. Unpaired *t*-test was run using GraphPad (version 5.00). Three biological replicates with two technical replicates each were performed. *P. berghei* sporozoites (*Pb*WT and *Pb*ΔPoFUT2) were collected 21–24 days post-feeding from infected *An. stephensi* females bred at Instituto de Medicina Molecular. Salivary glands were dissected and kept in non-supplemented RPMI media at 4°C. Salivary glands were then smashed in a microcentrifuge tube. To eliminate mosquito debris and isolate sporozoites, samples were filtered using a 70 μm strainer. Sporozoites were counted in a Neubauer chamber using an Olympus CKX41 inverted microscope. Six experiments were performed using GLMM for statistical analysis.

### *P. berghei* Gliding Motility Assays

For gliding motility analysis, 20,000 *P. berghei* sporozoites isolated from infected mosquito salivary glands were deposited on glass slides coated with anti-CSP monoclonal antibody (3D11 mouse mAb, 10 μg/mL final concentration) and incubated at 37°C for 1 h. Sporozoites were subsequently fixed in 4% paraformaldehyde for 10 min and blocked with 1% bovine serum albumin (BSA) in PBS for 1 h at RT. Parasites were stained with 3D11 antibody (10 μg/mL) for 1 h at RT, followed by three PBS washes. Sporozoites were further incubated (1:250) with goat anti-mouse AlexaFluor 488 (Jackson ImmunoResearch Laboratories). Three additional PBS washes were carried out. Sporozoites associated with CSP trails were visualized by fluorescence microscopy using a Zeiss Axiovert 200M microscope. Quantification was performed by counting the number of sporozoites performing < 3 circles, ≥ 3 and ≤ 10 circles, and > 10 circles.

### Human Hepatoma Cell Culture and *in vitro* Infection With *P. berghei* Parasites

Human hepatoma Huh7 cells were cultured in RPMI 1640 media supplemented with 10% v/v fetal bovine serum, 0.1 mM non-essential amino acids, 50 μg/mL penicillin/streptomycin, 2 mM glutamine, and 1 mM HEPES (final concentrations) at pH 7 and maintained at 37°C with 5% CO_2_.

For *in vitro* hepatic infections, cells were seeded on glass coverslips the day before infection. Sporozoite addition was followed by 5 min centrifugation at 1,800 × g. 48 h post-infection, cells were fixed for 20 min at RT and incubated with permeabilization/blocking solution (0.1% v/v Triton X-100, 1% w/v BSA in PBS) for 30 min at RT. Parasites were stained with an anti-UIS4 antibody (dilution 1:1,000, SICGEN # AB0042-200) for 1 h at RT, washed three times, and further incubated with anti-mouse AlexaFluor 488 secondary antibody (1:400 dilution) in the presence of Hoechst 33258 (1:1,000 dilution, Thermo Fisher Scientific) for nuclear staining. Coverslips were mounted on microscope slides with Fluoromount (SouthernBiotech). Widefield images for size determination were acquired with a Zeiss Axiovert 200M microscope. Images were processed with ImageJ software (version 1.47).

Sporozoite traversing activity was examined using a standard cell-wounding and membrane repair assay. Huh7 cells (1.0 × 10^4^ per well) were seeded in 96-well plates the day before infection. Sporozoites were added to cells for 2 h in the presence of 0.5 mg/mL FITC-labeled dextran (Thermo Fisher Scientific). Cells were collected for flow cytometry analysis 48 h post-infection and analyzed on a Becton Dickinson LSR Fortessa flow cytometer with the DIVA software (version 6.2). Analysis was carried out using the FlowJo software (version 6.4.7, FlowJo).

### *P. berghei* Sporozoite Infectivity to Mice

For transmission experiments, naïve C57BL/6 mice were exposed to 10 mosquitoes infected with *Pb*WT or *Pb*ΔPoFUT2 for 30 min; 5 mice were used per parasite clone. Parasitemias were followed daily by counting Giemsa stained smears from a drop of tail blood. Animals were monitored daily for clinical signs of cerebral malaria (head deviation, convulsions, ataxia, and paraplegia). Mice were euthanized when required, according to the approved protocol, to avoid further stress and pain.

### Statistical Analyses

GLMM was applied as described previously (Churcher et al., 2012) to test for differences between the prevalence of oocysts (assuming a binomial distribution error structure) and the average number of oocysts per mosquito (assuming a negative binomial distribution) (Bolker et al., 2012; Churcher et al., 2012). GraphPad Prism version 6 was used for standard statistical analyses as indicated in the respective sections above.

## Results

### Creation of *Plasmodium* PoFUT2 Null Mutants (*Pf*ΔPoFUT2 and *Pb*ΔPoFUT2)

To study the function of PoFUT2 in *Plasmodium* parasites, we knocked out this gene in the human malaria parasite *P. falciparum*. *P. falciparum* PoFUT2 (*Pf*PoFUT2) was disrupted by double crossover recombination using a targeting construct (pCC1-*Pf*PoFUT2; Maier et al., 2006) that replaced the gene with a *hu-dhfr* selection cassette in the *P. falciparum* NF54 line, the same parasite line used by Lopaticki et al. (Lopaticki et al., 2017). To add robustness to our experimental data, a similar approach was used to ablate *P. berghei* PoFUT2 (*Pb*PoFUT2) in ANKA strain 2.34 parasites, using the available PlasmoGEM resource PbGEM-283938 plasmid (Pfander et al., 2011). After cloning *hu-dhfr* resistant parasites by limiting dilution, PCR or Southern blot analyses confirmed PoFUT2 disruption in two clones from both *Plasmodium* species (Figure 1). As reported (Lopaticki et al., 2017), the ability to produce PoFUT2 null-mutant parasites demonstrates that the gene is not essential for asexual blood stage growth in culture or in a rodent model of infection.

### Oocyst Infection of Mosquito Midguts Is Similar in *Pf*ΔPoFUT2 and *Pf*WT

Considering the importance of TSR domain-containing proteins such as CTRP, CSP, and TRAP in the mosquito stages of parasite development, we examined the function of PoFUT2 throughout the complete life cycle of *Plasmodium*. Notably, CTRP is an ookinete surface protein containing 7 TSR domains and 5 conserved *O*-fucosylation motifs that plays a critical role in *Plasmodium* invasion of the *Anopheles* midgut (Dessens et al., 1999). To understand the importance of PoFUT2 for oocyst infection, we infected *An. gambiae* mosquitoes with *Pf*WT and *Pf*ΔPoFUT2 gametocyte cultures. Recognizing the inherent variation from experiment to experiment when using SMFA for mosquito infections (Churcher et al., 2012), we conducted 12 experiments to capture the entire variation in oocyst development in the mosquito midgut.

**Figure 2:**
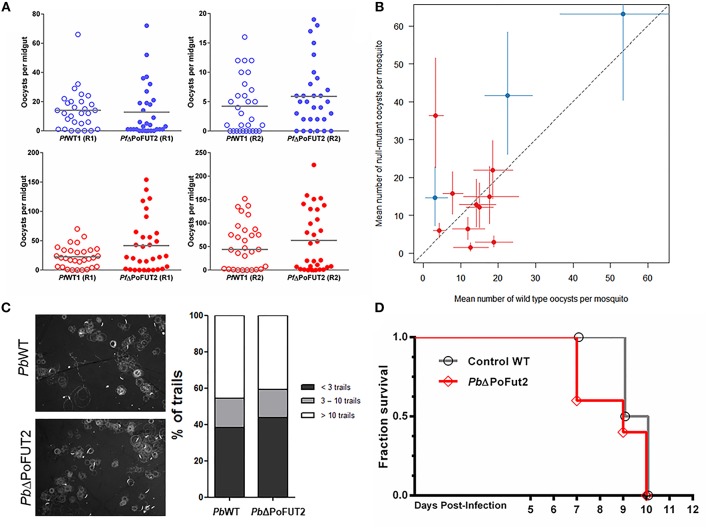
Wild type and PoFUT2 mutant *Plasmodium* parasites demonstrate comparable infectiousness to mosquitoes but exhibit species-specific difference in infection phenotypes for mosquito-mammal transmission. **(A)** *An. gambiae* female mosquitoes (*N* = 30 per group) were fed with *Pf*WT (open) and *Pf*ΔPoFUT2 (closed), and oocyst number per midgut was counted 8 days post-feed for two gametocyte concentrations: 0.03% (blue) and 0.3% (red). Two representative replicate experiments at each gametocyte concentration (R1, R2) are shown. Horizontal bars indicate the mean oocyst number per midgut. **(B)** Generalized Linear Mix Model statistical analysis of the mean oocyst intensity and infection prevalence from SMFAs performed with *Pf*WT or *Pf*ΔPoFUT2 in either 0.03% gametocytemia (blue) or 0.3% gametocytemia (red). Black dotted line indicates no difference between the two groups of mosquitoes; colored vertical, and horizontal lines denote 95% confidence intervals for the point estimates. **(C)** Motility assays were performed for *Pb*WT and *Pb*ΔPoFUT parasites to monitor CSP trails (left panels). The number of trails for each sporozoite was classified as < 3 circles (dark gray), 3–10 circles (light gray), or > 10 circles (white; right panel). **(D)** C57BL/6 mice were infected by mosquito direct feeding with *Pb*WT (gray line) or *Pb*ΔPoFUT2 (red line) and monitored for patency (blood stage infection) and subsequent survival.

We quantified the *P. falciparum* oocyst numbers on the *An. gambiae* midgut wall following *Pf*WT or *Pf*ΔPoFUT2 parasite infection (Figure 2A). GLMM was used to test for differences between the average number of oocysts (assuming a negative binomial distribution) and oocyst prevalence (assuming a binomial distribution; Churcher et al., 2012). An average difference between *Pf*WT and *Pf*ΔPoFUT2 parasites was calculated by including replicate number as a random effect, which accounts for differences in infectivity between the replicates. There was no significant difference in *P. falciparum* oocyst intensity (*P* value = 0.517) and prevalence (*P* value = 0.963) (Figure 2B). Furthermore, the diameter and mean size of *P. falciparum* oocysts were comparable for *Pf*WT and *Pf*ΔPoFUT2 parasites (Supplementary Figures 1A–D). Six biological replicates (with two technical replicates each) were performed with *P. falciparum* parasites.

### PoFUT2 Is Not Required for *An. gambiae* Salivary Gland Infection by Sporozoites

CSP is an important sporozoite surface protein with a single *O*-fucosylation site in the TSR domain. We quantified the number of sporozoites per salivary gland pair at day 14 post-blood feeding for *P. falciparum*. Three biological replicates (with two technical replicates each for a total of 6 experiments) were performed with *P. falciparum* (Supplementary Figure S2A). Using a GLMM approach, we observed that *Anopheles* mosquitoes carrying null-mutant or wild type parasites showed comparable numbers of salivary gland sporozoites (intensity counts *P* value = 0.446, prevalence *P* value = 0.645). Hence, PoFUT2 does not play a critical role for parasite maturation in oocysts or salivary gland colonization.

### PoFUT2 Is Not Essential in a Murine Malaria Model

To examine the cross-species role of PoFUT2, additional functional assays were carried out with *P. berghei* parasites lacking PoFUT2 (*Pb*ΔPoFUT2). The murine malaria model allows for the use of non-transgenic mice, which are infected routinely by *P. berghei* sporozoites. We infected *An. stephensi* mosquitoes with *Pb*WT and *Pb*ΔPoFUT2 parasites and examined oocyst intensity and prevalence, salivary gland sporozoite characteristics, and *in vivo* infection of mice. In the *P. berghei*-*An. stephensi* model, there was no significant difference in *P. berghei* oocyst intensity (*P* value = 0.656) and prevalence (*P* value = 0.962); which corroborated our *P. falciparum* results in *An. gambiae*

Sporozoite motility assays (Figure 2C) and *in vitro* hepatic infection experiments with *Pb*ΔPoFUT2 mutant parasites (Supplementary Figures 2B,C) produced comparable results between WT and null-mutant parasites. Finally, two independent experiments performed with *P. berghei* showed that *Pb*ΔPoFUT2 sporozoites were able to infect C57BL/6 mice in mosquito “bite-back” direct feeding experiments (Figure 2D). For *Pb*WT (gray lines, Figure 2D), 4/5 mice succumbed to cerebral malaria (2 at day 9 and 2 at day 10) and the fifth mouse that remained alive with high parasitemia was sacrificed on day 10. For *Pb*ΔPoFUT2 (red lines, Figure 2D), 4/5 mice succumbed to cerebral malaria (2 at day 7, 1 at day 9, and 1 at day 10) and the fifth mouse that remained alive with high parasitemia was sacrificed on day 10. Comparable sporozoite numbers developed in mosquitoes for both *Pb*WT and *Pb*ΔPoFUT2 (Table 1), akin to what was observed for *P. falciparum* (Supplementary Figure 2A). The apparent reduction in sporozoites/salivary gland pair in Experiment A is not reproducible (as shown in Experiment B), underscoring the variation in the system. These results demonstrated that *Pb*ΔPoFUT2 mutants are fully infectious and able to progress through skin passage, hepatocyte infection, and intrahepatic or liver stage development.

**Table 1:** *Plasmodium berghei* WT and *Pb*ΔPoFUT2 “bite back” infections in mice.

**Expt**	***Pb*WT (N)**	**% Infected mice (n/N)**	***Pb*ΔPoFUT2 (N)**	**% Infected mice (n/N)**
A	114,000/mosquito (13)	100% (5/5)	65,019/mosquito (13)	100% (5/5)
B	133,750/mosquito (10)	100% (5/5)	148,255/mosquito (10)	100% (5/5)

## Discussion

*O*-fucosylation, the process of adding a fucose residue to the -OH side chain of serines or threonines in TSR cysteine-rich domains (Leonhard-Melief and Haltiwanger, 2010), is mediated by PoFUT2 *O*-fucosyltransferase (Valero-González et al., 2016). The *O*-fucosylation of *P. falciparum* CSP and TRAP sporozoite proteins has been recently demonstrated (Swearingen et al., 2016). Considering the relevance of these and other TSR domain-containing proteins for *Plasmodium* invasion and motility (Morahan et al., 2009), we created a PoFUT2 null-mutant in human and rodent parasites to completely assess the effect of PoFUT2 disruption throughout the parasite life cycle. We observed that PoFUT2 was not required for either *P. falciparum* or *P. berghei* infection of *Anopheles* mosquitoes by counting the number of oocysts per midgut. These data are in clear contrast to the reported observation by Lopaticki et al. (2017), which pooled data from replicate assays. Pooling data without statistical support may generate misleading results, as there can be large variability in average oocyst counts between replicates (as seen in Figure 2B) with single studies biasing overall estimates. However, testing differences using robust and adequate GLMMs is more appropriate to analyze SMFA assays (Churcher et al., 2012), which have a high variability risk due to the use of different sources of blood, cages, or feeders and the generation of highly dispersed data. GLMM, a multi-level analysis enabling the estimation of a fixed effect (number of oocysts) while allowing the level of infection in different assays to vary at random, is the statistical model of choice for assessing parasite infectiousness to mosquito vectors (Churcher et al., 2015; Kapulu et al., 2015).

Remarkably, our primary parasite-mosquito model pits an African parasite (NF54) with an African mosquito vector (*An. gambiae* KEELE), which represents the closest natural combination in laboratory studies. *An. stephensi* (SDA-500) has also been the customary laboratory vector for *P. berghei* studies. Although other groups have used *An. stephensi*-*P. falciparum* (NF54) combinations, we have found that the overall mean oocyst intensity tends to be lower than what is observed for *An. gambiae-P. falciparum*. We tested two gametocyte densities of 0.03 and 0.3% to determine whether any potential phenotypes would be observed at lower parasite densities during mosquito infection. In both cases, no reductions in oocyst development were observed. Therefore, in our hands PoFUT2 disruption did not affect oocyst development in the most relevant parasite-mosquito species combinations in a laboratory setting for both human and murine malaria models.

CSP and TRAP are important sporozoite TSR domain-containing proteins which are *O*-fucosylated by PoFUT2 (Swearingen et al., 2016). However, *Plasmodium* PoFUT2 null-mutants and wild type parasites showed comparable numbers of sporozoites in mosquito salivary glands. Furthermore, sporozoite motility, and infectivity was similar in *Pb*WT and *Pb*ΔPoFUT2 parasites, in agreement with mouse infectivity data. Interestingly, TRAP secretion appears to have been partly affected in the work published by Lopaticki et al. (2017). This is not surprising, as it is known that the intensity of attenuated PoFUT2-ablation phenotypes are protein-specific (Vasudevan et al., 2015). However, the ookinete and sporozoite infectivity in mosquitoes and vertebrates, respectively, remains unchanged; indicating that functional redundancy or the presence of multiple parasite “invasins” ensures the success of the malaria parasite during transmission. As Lopaticki et al. (2017), mention in their study, the absence of many genes necessary for classical glycosylation in *Plasmodium* genomes (von Itzstein et al., 2008; Cova et al., 2015), together with some contradictory results (Kimura et al., 1996; Gowda et al., 1997), fueled the debate about the existence of protein glycosylation in the malaria parasite, with the exception of glycosylphosphatidylinositol anchors (Naik et al., 2000). Recently, studies are casting new light on this issue (Bushkin et al., 2010; Sanz et al., 2013, 2016; Swearingen et al., 2016; López-Gutiérrez et al., 2017). Additionally, recent work exploring the function of PoFUT2 in *Toxoplasma gondii*, a *Plasmodium* related parasite, also revealed discrepancies in the effect of PoFUT2 null mutants on microneme protein 2 secretion and host cell attachment and invasion (Bandini et al., 2019; Gas-Pascual et al., 2019; Khurana et al., 2019). Our results suggest that within *Plasmodia*, nuanced differences with respect to developmental biology and host preference can also result in the observation of diverse phenotypes, which in this case is specific to *O*-fucosylation of sporozoite TSR domain-containing proteins. Taking into account previous and current controversies about the glycobiology of *Plasmodium* and our experimental results reported here, we note that caution must be exercised before considering protein *O*-fucosylation as a strict requirement for the efficient infection of mosquito and vertebrate hosts for all *Plasmodium* species.

## Data Availability

All datasets generated for this study are included in the manuscript and/or the Supplementary Files.

## Author Contributions

SS, RD, and LI conceived the work. SS and EA knocked out and genotyped *Plasmodium falciparum* and *Plasmodium berghei* lines, respectively. SS, RD, RT, AT, BH, TH, and GV conducted the oocyst and sporozoite experiments with *P. falciparum* strains. EA, MM, and JR were responsible for *P. berghei* experiments. SS, LI, and RD outlined the manuscript. TC and RD performed SMFAs GLMM statistical analyses. All authors contributed to the writing and review of this manuscript.

### Conflict of Interest Statement

The authors declare that the research was conducted in the absence of any commercial or financial relationships that could be construed as a potential conflict of interest.

## Supplementary Material

Supplementary Figure 1 Oocyst development as a proxy-measure for successful ookinete invasion of the mosquito midgut epithelium is not impacted in *Pf*ΔPoFUT2 parasites. **(A,B)** Brightfield images of mercurochrome-stained *Pf*WT or *Pf*ΔPoFUT2 oocysts. **(C,D)** The range of *Plasmodium falciparum* oocyst diameters within individually mosquitoes (μm) **(C)** and the mean diameter of oocysts for all infected mosquitoes (μm) **(D)** of the *Pf*WT (white) and *Pf*ΔPoFUT2 (gray) oocyst is represented. Unpaired *t*-test *P* value = 0.6897. The horizontal bar indicates the mean oocyst diameter.

Supplementary Figure 2 Sporozoite development, motility, and infection of hepatocytes by null-mutant parasites is unaffected. **(A)** *P. falciparum* sporozoites (*Pf*WT and *Pf*ΔPoFUT2) were counted on day 14 post-blood feeding. Two representative experiments of six total are shown. **(B)** *in vitro* hepatocyte infection of Huh7 cells normalized to *Pb*WT infection as 100%. Mann-Whitney *U* test *P* value = 0.1716. **(C)** Exoerythrocytic form (EEF) size in Huh7 hepatocyte cell infection model with *Pb*WT and *Pb*ΔPoFUT2 sporozoites. Unpaired *t*-test *P* value = 0.3931.

## References

[B1] Adams J. C. Tucker R. P. (2000). The thrombospondin type 1 repeat (TSR) superfamily: diverse proteins with related roles in neuronal development. Dev. Dyn. 218, 280–299. 10.1002/(SICI)1097-0177(200006)218:2<280::AID-DVDY4>3.0.CO;2-0 10842357

[B2] Bandini G. Leon D. R. Hoppe C. M. Zhang Y. Agop-Nersesian C. Shears M. J.. (2019). O-Fucosylation of thrombospondin-like repeats is required for processing of microneme protein 2 and for efficient host cell invasion by *Toxoplasma gondii* tachyzoites. J. Biol. Chem. 294, 1967–1983. 10.1074/jbc.RA118.005179 30538131 PMC6369279

[B3] Bolker B. Skaug H. Magnusson A. Nielsen A. (2012). Getting Started With the glmmADMB Package. Available online at: http://glmmadmb.r-forge.r-project.org/glmmADMB.pdf

[B4] Bushkin G. G. Ratner D. M. Cui J. Banerjee S. Duraisingh M. T. Jennings C. V.. (2010). Suggestive evidence for Darwinian selection against asparagine-linked glycans of *Plasmodium falciparum* and *Toxoplasma gondii*. Eukaryot. Cell 9, 228–241. 10.1128/EC.00197-09 19783771 PMC2823003

[B5] Chattopadhyay R. Rathore D. Fujioka H. Kumar S. de la Vega P. Haynes D.. (2003). PfSPATR, a *Plasmodium falciparum* protein containing an altered thrombospondin type I repeat domain is expressed at several stages of the parasite life cycle and is the target of inhibitory antibodies. J. Biol. Chem. 278, 25977–25981. 10.1074/jbc.M300865200 12716913

[B6] Churcher T. S. Blagborough A. M. Delves M. Ramakrishnan C. Kapulu M. C. Williams A. R.. (2012). Measuring the blockade of malaria transmission–an analysis of the standard membrane feeding assay. Int. J. Parasitol. 42, 1037–1044. 10.1016/j.ijpara.2012.09.002 23023048

[B7] Churcher T. S. Trape J. F. Cohuet A. (2015). Human-to-mosquito transmission efficiency increases as malaria is controlled. Nat. Commun. 6:6054. 10.1038/ncomms7054 25597498 PMC4309425

[B8] Coppi A. Natarajan R. Pradel G. Bennett B. L. James E. R. Roggero M. A.. (2011). The malaria circumsporozoite protein has two functional domains, each with distinct roles as sporozoites journey from mosquito to mammalian host. J. Exp. Med. 208, 341–356. 10.1084/jem.20101488 21262960 PMC3039851

[B9] Cova M. Rodrigues J. A. Smith T. K. Izquierdo L. (2015). Sugar activation and glycosylation in Plasmodium. Malar. J. 14:427. 10.1186/s12936-015-0949-z 26520586 PMC4628283

[B10] Dessens J. T. Beetsma A. L. Dimopoulos G. Wengelnik K. Crisanti A. Kafatos F. C.. (1999). CTRP is essential for mosquito infection by malaria ookinetes. EMBO J. 18, 6221–6227. 10.1093/emboj/18.22.6221 10562534 PMC1171685

[B11] Gas-Pascual E. Ichikawa H. T. Sheikh M. O. Serji M. I. Deng B. Mandalasi M.. (2019). CRISPR/Cas9 and glycomics tools for *Toxoplasma* glycobiology. J. Biol. Chem. 294, 1104–1125. 10.1074/jbc.RA118.006072 30463938 PMC6349120

[B12] Gowda D. C. Gupta P. Davidson E. A. (1997). Glycosylphosphatidylinositol anchors represent the major carbohydrate modification in proteins of intraerythrocytic stage *Plasmodium falciparum*. J. Biol. Chem. 272, 6428–6439. 10.1074/jbc.272.10.6428 9045667

[B13] Janse C. J. Ramesar J. Waters A. P. (2006). High-efficiency transfection and drug selection of genetically transformed blood stages of the rodent malaria parasite *Plasmodium berghei*. Nat. Protoc. 1, 346–356. 10.1038/nprot.2006.53 17406255

[B14] Kapulu M. C. Da D. F. Miura K. Li Y. Blagborough A. M. Churcher T. S.. (2015). Comparative assessment of transmission-blocking vaccine candidates against *Plasmodium falciparum*. Sci. Rep. 5:11193. 10.1038/srep11193 26063320 PMC4463016

[B15] Khurana S. Coffey M. J. John A. Uboldi A. D. Huynh M. H. Stewart R. J.. (2019). Protein O-fucosyltransferase 2–mediated O-glycosylation of the adhesin MIC2 is dispensable for *Toxoplasma gondii* tachyzoite infection. J. Biol. Chem. 294, 1541–1553. 10.1074/jbc.RA118.005357 30514763 PMC6364784

[B16] Kimura E. A. Couto A. S. Peres V. J. Casal O. L. Katzin A. M. (1996). N-linked glycoproteins are related to schizogony of the intraerythrocytic stage in *Plasmodium falciparum*. J. Biol. Chem. 271, 14452–14461. 10.1074/jbc.271.24.14452 8662869

[B17] Kozma K. Keusch J. J. Heggemann B. Luther K. B. Klein D. Hess D.. (2006). Identification and characterization of abeta1,3-glucosyltransferase that synthesizes the Glc-beta1,3-Fuc disaccharide on thrombospondin type 1 repeats. J. Biol. Chem. 281, 36742–36751. 10.1074/jbc.M605912200 17032646

[B18] Leonhard-Melief C. Haltiwanger R. S. (2010). O-Fucosylation of thrombospondin type 1 repeats. Methods Enzymol. 480, 401–416. 10.1016/S0076-6879(10)80018-7 20816219

[B19] Li F. Templeton T. J. Popov V. Comer C. E. Tsuboi T. Torii M.. (2004). *Plasmodium* ookinete-secreted proteins secreted through a common micronemal pathway are targets of blocking malaria transmission. J. Biol. Chem. 279, 26635–26644. 10.1074/jbc.M401385200 15069061

[B20] Lopaticki S. Yang A. S. P. John A. Scott N. E. Lingford J. P. O'Neill M. T.. (2017). Protein O-fucosylation in *Plasmodium falciparum* ensures efficient infection of mosquito and vertebrate hosts. Nat. Commun. 8:561. 10.1038/s41467-017-00571-y 28916755 PMC5601480

[B21] López-Gutiérrez B. Dinglasan R. R. Izquierdo L. (2017). Sugar nucleotide quantification by liquid chromatography tandem mass spectrometry reveals a distinct profile in *Plasmodium falciparum* sexual stage parasites. Biochem. J. 474, 897–905. 10.1042/BCJ20161030 28104756 PMC5340172

[B22] Luo Y. Nita-Lazar A. Haltiwanger R. S. (2006). Two distinct pathways for O-fucosylation of epidermal growth factor-like or thrombospondin type 1 repeats. J. Biol. Chem. 281, 9385–9392. 10.1074/jbc.M511974200 16464858

[B23] Maier A. G. Braks J. M. Waters A. P. Cowman A. F. (2006). Negative selection using yeast cytosine deaminase/uracil phosphoribosyl transferase in *Plasmodium falciparum* for targeted gene deletion by double crossover recombination. Mol. Biochem. Parasitol. 150, 118–121. 10.1016/j.molbiopara.2006.06.014 16901558

[B24] Mathias D. K. Pastrana-Mena R. Ranucci E. Tao D. Ferruti P. Ortega C.. (2013). A small molecule glycosaminoglycan mimetic blocks *Plasmodium* invasion of the mosquito midgut. PLoS Pathog. 9:e1003757. 10.1371/journal.ppat.1003757 24278017 PMC3836724

[B25] Menard R. Tavares J. Cockburn I. Markus M. Zavala F. Amino R. (2013). Looking under the skin: the first steps in malarial infection and immunity. Nat. Rev. Microbiol. 11, 701–12. 10.1038/nrmicro3111 24037451

[B26] Moon R. W. Hall J. Rangkuti F. Ho Y. S. Almond N. Mitchell G. H.. (2013). Adaptation of the genetically tractable malaria pathogen *Plasmodium knowlesi* to continuous culture in human erythrocytes. Proc. Natl. Acad. Sci. U. S. A. 110, 531–536. 10.1073/pnas.1216457110 23267069 PMC3545754

[B27] Moorthy V. S. Imoukhuede E. B. Milligan P. Bojang K. Keating S. Kaye P.. (2004). A randomised, double-blind, controlled vaccine efficacy trial of DNA/MVA ME-TRAP against malaria infection in Gambian adults. PLoS Med. 1:e33. 10.1371/journal.pmed.0010033 15526058 PMC524376

[B28] Morahan B. J. Wang L. Coppel R. L. (2009). No TRAP, no invasion. Trends Parasitol. 25, 77–84. 10.1016/j.pt.2008.11.004 19101208

[B29] Naik R. S. Branch O. H. Woods A. S. Vijaykumar M. Perkins D. J. Nahlen B. L.. (2000). Glycosylphosphatidylinositol anchors of Plasmodium falciparum: molecular characterization and naturally elicited antibody response that may provide immunity to malaria pathogenesis. J. Exp. Med. 192, 1563–1576. 10.1084/jem.192.11.1563 11104799 PMC2193092

[B30] Pfander C. Anar B. Schwach F. Otto T. D. Brochet M. Volkmann K.. (2011). A scalable pipeline for highly effective genetic modification of a malaria parasite. Nat. Methods 8, 1078–1082. 10.1038/nmeth.1742 22020067 PMC3431185

[B31] Ricketts L. M. Dlugosz M. Luther K. B. Haltiwanger R. S. Majerus E. M. (2007). O-fucosylation is required for ADAMTS13 secretion. J. Biol. Chem. 282, 17014–17023. 10.1074/jbc.M.700317200 17395589

[B32] RTS,S Clinical Trials Partnership (2015). Efficacy and safety of RTS,S/AS01 malaria vaccine with or without a booster dose in infants and children in Africa: final results of a phase 3, individually randomised, controlled trial. Lancet 386, 31–45. 10.1016/S0140-6736(15)60721-8 25913272 PMC5626001

[B33] Sanz S. Bandini G. Ospina D. Bernabeu M. Marino K. Fernandez-Becerra C.. (2013). Biosynthesis of GDP-fucose and other sugar nucleotides in the blood stages of *Plasmodium falciparum*. J. Biol. Chem. 288, 16506–16517. 10.1074/jbc.M112.439828 23615908 PMC3675586

[B34] Sanz S. López-Gutiérrez B. Bandini G. Damerow S. Absalon S. Dinglasan R. R.. (2016). The disruption of GDP-fucose de novo biosynthesis suggests the presence of a novel fucose-containing glycoconjugate in *Plasmodium* asexual blood stages. Sci. Rep. 6:37230. 10.1038/srep37230 27849032 PMC5110956

[B35] Swearingen K. E. Lindner S. E. Shi L. Shears M. J. Harupa A. Hopp C. S.. (2016). Interrogating the *Plasmodium* sporozoite surface: identification of surface-exposed proteins and demonstration of glycosylation on CSP and TRAP by mass spectrometry-based proteomics. PLoS Pathog. 12:e1005606. 10.1371/journal.ppat.1005606 27128092 PMC4851412

[B36] Tan K. Duquette M. Liu J. H. Dong Y. Zhang R. Joachimiak A.. (2002). Crystal structure of the TSP-1 type 1 repeats: a novel layered fold and its biological implication. J. Cell Biol. 159, 373–382. 10.1083/jcb.200206062 12391027 PMC2173040

[B37] Trager W. Jensen J. B. (1976). Human malaria parasites in continuous culture. Science 193, 673–675. 10.1126/science.781840 781840

[B38] Valero-González J. Leonhard-Melief C. Lira-Navarrete E. Jiménez-Osés G. Hernández-Ruiz C. Pallarés M. C.. (2016). A proactive role of water molecules in acceptor recognition by protein O-fucosyltransferase 2. Nat. Chem. Biol. 12, 240–246. 10.1038/nchembio.2019 26854667 PMC4845761

[B39] Vasudevan D. Haltiwanger R. S. (2014). Novel roles for O-linked glycans in protein folding. Glycoconj. J. 31, 417–426. 10.1007/s10719-014-9556-4 25186198 PMC4214879

[B40] Vasudevan D. Takeuchi H. Johar S. S. Majerus E. Haltiwanger R. S. (2015). Peters plus syndrome mutations disrupt a noncanonical ER quality-control mechanism. Curr. Biol. 25, 286–295. 10.1016/j.cub.2014.11.049 25544610 PMC4318717

[B41] von Itzstein M. Plebanski M. Cooke B. M. Coppel R. L. (2008). Hot, sweet and sticky: the glycobiology of *Plasmodium falciparum*. Trends Parasitol. 24, 210–218. 10.1016/j.pt.2008.02.007 18420458

[B42] Wang L. W. Dlugosz M. Somerville R. P. Raed M. Haltiwanger R. S. Apte S. S. (2007). O-fucosylation of thrombospondin type 1 repeats in ADAMTS-like-1/punctin-1 regulates secretion: implications for the ADAMTS superfamily. J. Biol. Chem. 282, 17024–17031. 10.1074/jbc.M701065200 17395588

[B43] Wengelnik K. Spaccapelo R. Naltaz S. Robson K. J. Janse C. J. Bistoni F.. (1999). The A-domain and the thrombospondin-related motif of *Plasmodium falciparum* TRAP are implicated in the invasion process of mosquito salivary glands. EMBO J. 18, 5195–5204. 10.1093/emboj/18.19.5195 10508153 PMC1171590

[B44] WHO (2018). World Malaria Report 2018. Geneva, Switzerland: World Health Organization.

