# Supplementary figures and images for: Protein O-Fucosyltransferase 2 Is Not Essential for Plasmodium berghei Development
Source: Front Cell Infect Microbiol. 2019 Jul 3;9:238. doi: 10.3389/fcimb.2019.00238 (PMC6616114; doi:10.3389/fcimb.2019.00238)

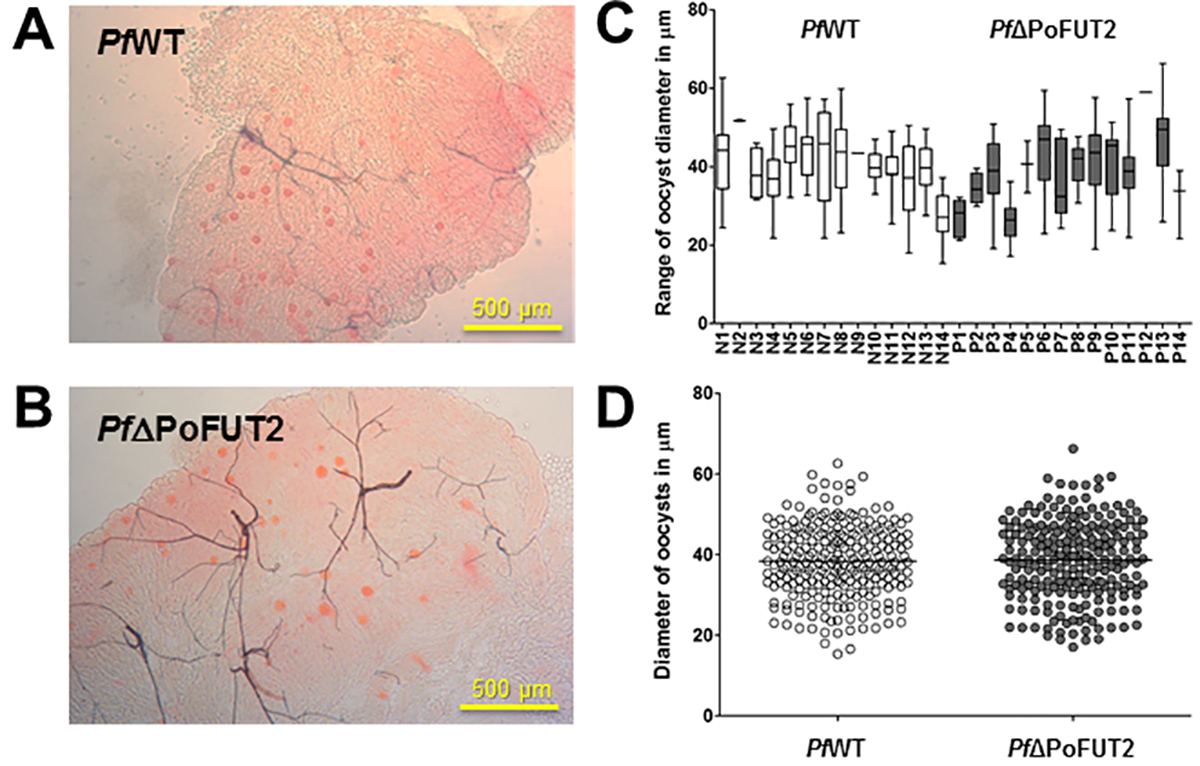

Supplement: Supplementary Figure 1 — Oocyst development as a proxy-measure for successful ookinete invasion of the mosquito midgut epithelium is not impacted in PfΔPoFUT2 parasites. (A,B) Brightfield images of mercurochrome-stained PfWT or PfΔPoFUT2 oocysts. (C,D) The range of Plasmodium falciparum oocyst diameters within individually mosquitoes (μm) (C) and the mean diameter of oocysts for all infected mosquitoes (μm) (D) of the PfWT (white) and PfΔPoFUT2 (gray) oocyst is represented. Unpaired t-test P value = 0.6897. The horizontal bar indicates the mean oocyst diameter. [file Image_1.TIF]

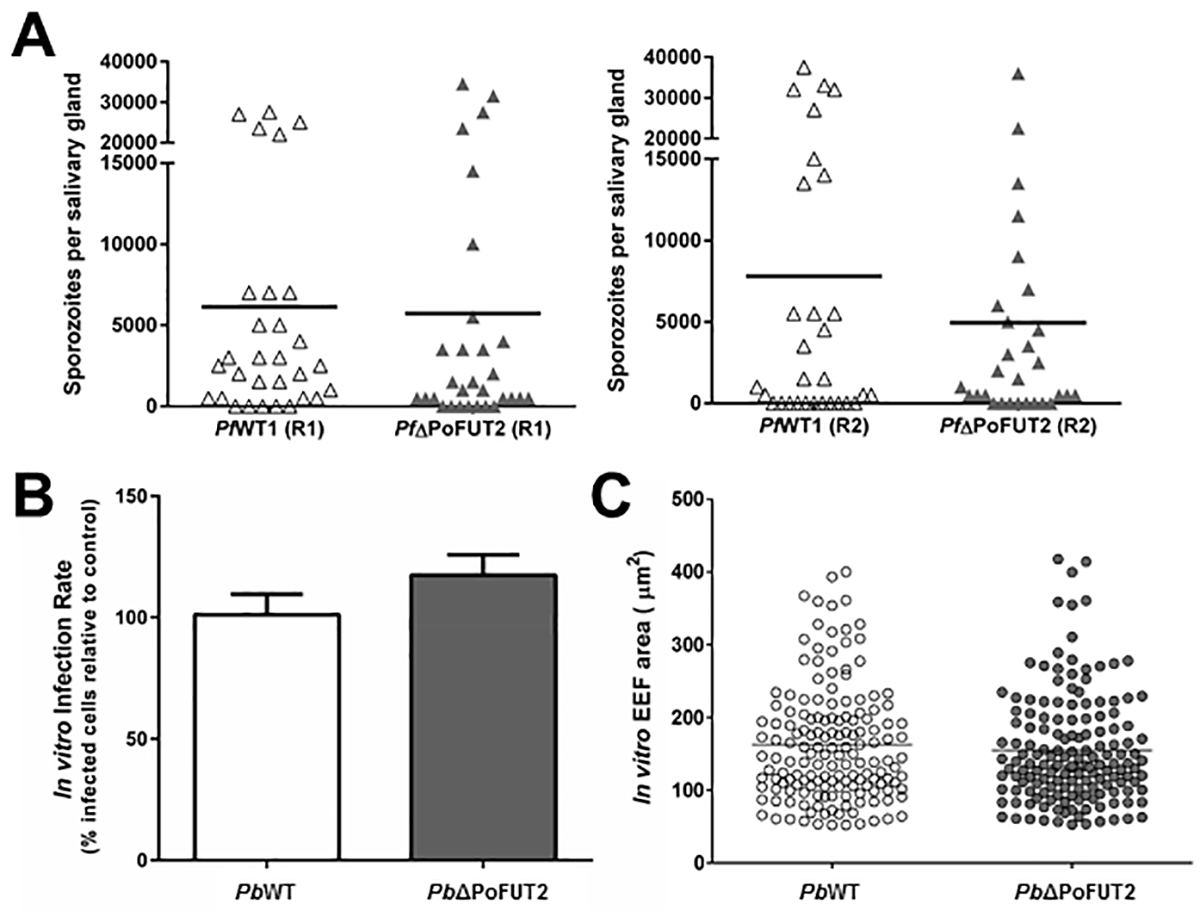

Supplement: Supplementary Figure 2 — Sporozoite development, motility, and infection of hepatocytes by null-mutant parasites is unaffected. (A) P. falciparum sporozoites (PfWT and PfΔPoFUT2) were counted on day 14 post-blood feeding. Two representative experiments of six total are shown. (B) in vitro hepatocyte infection of Huh7 cells normalized to PbWT infection as 100%. Mann-Whitney U test P value = 0.1716. (C) Exoerythrocytic form (EEF) size in Huh7 hepatocyte cell infection model with PbWT and PbΔPoFUT2 sporozoites. Unpaired t-test P value = 0.3931. [file Image_2.TIF]
